# Betulinic Acid Enhances the Viability of Random-Pattern Skin Flaps by Activating Autophagy

**DOI:** 10.3389/fphar.2019.01017

**Published:** 2019-09-13

**Authors:** Jiafeng Li, Guodong Bao, Eman ALyafeai, Jian Ding, Shihen Li, Shimin Sheng, Zitong Shen, Zhenyu Jia, Chen Lin, Chenxi Zhang, Zhiling Lou, Huazi Xu, Weiyang Gao, Kailiang Zhou

**Affiliations:** ^1^Department of Orthopaedics, The Second Affiliated Hospital and Yuying Children’s Hospital of Wenzhou Medical University, Wenzhou, China; ^2^Zhejiang Provincial Key Laboratory of Orthopaedics, Wenzhou, China; ^3^The Second Clinical Medical College of Wenzhou Medical University, Wenzhou, China; ^4^School of Pharmaceutical Science, Wenzhou Medical University, Wenzhou, China; ^5^Renji College of Wenzhou Medical University, Wenzhou, China

**Keywords:** betulinic acid, random-pattern skin flaps, autophagy, angiogenesis, apoptosis, oxidative stress

## Abstract

Random-pattern skin flap replantation is commonly used to repair skin defects during plastic and reconstructive surgery. However, flap necrosis due to ischemia and ischemia–reperfusion injury limits clinical applications. Betulinic acid, a plant-derived pentacyclic triterpene, may facilitate flap survival. In the present study, the effects of betulinic acid on flap survival and the underlying mechanisms were assessed. Fifty-four mice with a dorsal random flap model were randomly divided into the control, betulinic acid group, and the betulinic acid + 3-methyladenine group. These groups were treated with dimethyl sulfoxide, betulinic acid, and betulinic acid plus 3-methyladenine, respectively. Flap tissues were acquired on postoperative day 7 to assess angiogenesis, apoptosis, oxidative stress, and autophagy. Betulinic acid promoted survival of the skin flap area, reduced tissue edema, and enhanced the number of microvessels. It also enhanced angiogenesis, attenuated apoptosis, alleviated oxidative stress, and activated autophagy. However, its effects on flap viability and angiogenesis, apoptosis, and oxidative stress were reversed by the autophagy inhibitor 3-methyladenine. Our findings reveal that betulinic acid improves survival of random-pattern skin flaps by promoting angiogenesis, dampening apoptosis, and alleviating oxidative stress, which mediates activation of autophagy.

## Introduction

Random-pattern skin flap replantation has increasingly been used to repair skin defects or refractory wounds, damage from accidental trauma, cancer excisions, and diabetes mellitus *via* plastic and reconstructive surgery (Basu et al., 2014; Wang et al., 2014a; Park et al., 2015). However, flap necrosis, one of the most common postoperative complications that occurs during reconstructive surgery (Bai et al., 2018), restricts the length-to-width ratio of flaps to 1.5:2 (Milton, 1970; Cao et al., 2015). Blood supply to the flap is mainly supported by vasoganglion in the pedicle bed of the flap, and angiogenesis begins from the flap pedicle toward the distal part (Lin et al., 2017; Lin et al., 2018b). Consequently, the reason for severe distal flap necrosis is a deficient arterial blood supply and insufficient venous outflow (Kerrigan, 1983; Basu et al., 2014; Seyed Jafari et al., 2017). Furthermore, after neovascularization, restoration and reperfusion of the blood supply trigger ischemia–reperfusion injury (IRI) of ischemic flaps (Siemionow and Arslan, 2004; van den Heuvel et al., 2009). A burst of reactive oxygen species (ROS) is the chief culprit in the pathogenesis of IRI, accompanied by impaired cellular redox homeostasis and extensive cell apoptosis (Chu-Chiao and Bratton, 2013; Widgerow, 2014; Sies et al., 2017). Although several studies have reported that ROS have positive effects on neovascularization (De Barros et al., 2013; Huang and Nan, 2019; Yang, 2019), the majority of scholars hold the opinion that ROS accumulation and apoptosis of functional cells induced by IRI are the main factors resulting from necrosis of a skin flap (Gurlek et al., 2006; van den Heuvel et al., 2009; Ren et al., 2018). Considering these mechanisms, a number of treatments that involve promoting angiogenesis and attenuating oxidative stress and apoptosis have been used to improve survival of skin flaps.

Autophagy is an evolutionally conserved cell process that degrades cytosolic macromolecules and organelles to generate biomolecules depending on the lysosome (or vacuole). Autophagy is necessary for survival of cells under nutrient-deprived conditions (Glick et al., 2010; Du et al., 2012). The induction of autophagy promotes angiogenesis in bovine aortic endothelial cells (ECs), and ROS production and activation of AKT might be underlying mechanisms of angiogenesis (Glick et al., 2010). In ATP7B-deficient hepatocytes, such as in those in patients with Wilson disease, induction of autophagy responds to copper overload to prevent copper-induced apoptosis (Polishchuk et al., 2019). Remote ischemic preconditioning alleviates IRI of the liver by activating heme oxygenase 1 (HO-1)/p38-mitogen-activated protein kinase (MAPK)–dependent autophagy (Wang et al., 2014b), and bone marrow–derived mesenchymal stem cell–induced activation of autophagy *via* the phosphatidylinositol 3-kinase (PI3K)/Akt signaling pathway has been proven to protect the lung from IRI (Li et al., 2015b). Consequently, these studies suggest that the viability of skin flaps is enhanced by activating autophagy, promoting angiogenesis, and reducing apoptosis and oxidative stress.

Betulinic acid (BA) is a naturally occurring pentacyclic triterpene derived primarily from foods, medicinal herbs, and plants, particularly from white birch bark, and is commonly used to treat ischemic diseases (Lu et al., 2017; Liu et al., 2018; Wang and Zhao, 2018). The underlying mechanisms of BA involve promoting microcirculation by improving the microvascular reaction (Sarkaki et al., 2018). Betulinic acid protects against cerebral IRI by downregulating NADPH oxidase 4 in ischemic stroke (Lu et al., 2017). It inhibits oxidative stress and cell apoptosis by inducing Nrf2/HO-1 and inhibiting the p38 and JNK pathways in myocardial infarction (Wang et al., 2018) and elevates autophagy levels (Liu et al., 2018), which is beneficial for angiogenic function (Dai et al., 2018). Because random flaps are susceptible to ischemia, we hypothesized that BA promotes the survival of flaps through the above mechanisms. In this study, the therapeutic actions of BA in skin flaps were explored, as well as the underlying mechanisms.

## Materials and Methods

### Animals

C57BL/6 mice (male, 20–30 g) were acquired from the laboratory animal center of Wenzhou Medical University (license no. SCXK [ZJ] 2005-0019). Surgery, treatments, and postoperative care strictly followed the Guide for the Care and Use of Laboratory Animals of the China National Institutes of Health. All procedures involving mice were carried out with approval of the Animal Research Committee of Wenzhou Medical University (wydw2017-0022). Best efforts were made to minimize the pain of the mice. All mice in this experiment were housed individually in standard experimental cages, with a 12-h light/dark cycle and free access to regular food and water before any experimental procedure. Fifty-four mice were randomly divided into the control, BA, and BA + 3-methyladenine (3MA) groups (n = 18 each).

### Reagents and Antibodies

BA (C_3_0H_48_O_3_, purity ≥98%) was purchased from MedChemExpress (Shanghai, China). Diaminobenzidine (DAB) developer, pentobarbital sodium, and the hematoxylin and eosin (H&E) staining kit were provided by Solarbio Science & Technology (Beijing, China). Rabbit monoclonal anti–cadherin 5 was purchased from Boster Biological Technology (A02632–2; Wuhan, China). Rabbit monoclonal anti-GAPDH was acquired from Biogot Technology (AP0063; Shanghai, China). The rabbit monoclonal anti–vascular endothelial growth factor (VEGF), anti–superoxide dismutase 1 (SOD1), anti–phosphoinositide-3-kinase (VPS34), anti–matrix metalloproteinase 9 (MMP9), anti-HO1, anti–cathepsin D (CTSD), and anti-CAPS3 were acquired from Proteintech Group (19003-1, 10269-1, 12452-1, 10375-2, 10701-1, 21327-1, and 19677-1; Chicago, IL, USA). Rabbit monoclonal anti–endothelial nitric oxide synthase (eNOS), anti–cytochrome *c* (CYC), and anti-Bax were purchased from Cell Signaling Technology (12994, 14796, and 32027; Beverly, MA, USA). Mouse monoclonal anti–SQSTM1/p62 was purchased from Abcam (ab56416; Cambridge, UK). Rabbit monoclonal anti–microtubule-associated 1 protein light chain 3 (LC3) and 3MA were purchased from Sigma-Aldrich Chemical Co. (L7543 and M9281; Milwaukee, WI, USA). Horseradish peroxidase (HRP)–conjugated immunoglobulin G (IgG) secondary antibody was provided by Santa Cruz Biotechnology (Dallas, TX, USA). Fluorescein isothiocyanate (FITC)–conjugated IgG secondary antibody was obtained from Boyun Biotechnology (Nanjing, China), and the 4′,6-diamidino-2-phenylindole (DAPI) solution was purchased from Beyotime Biotechnology (Jiangsu, China). The Electrochemiluminescence (ECL) Plus Reagent Kit was obtained from PerkinElmer Life Sciences (Waltham, MA, USA) and the BCA kit was acquired from ThermoFisher Scientific (Rockford, IL, USA).

### Random-Pattern Skin Flap Model

Before the operation, 50 mg/kg pentobarbital sodium 1% (w/v) was injected intraperitoneally to anesthetize the mice. Then a caudal-based skin/panniculus carnosus flap (size 1.5 × 4.5 cm^2^) was elevated on the mouse dorsum beneath the fascia using a mouse dorsal random flap model as reported previously (Lee et al., 2017). Next, the right and left sacral arteries supporting the blood supply of this flap were excised completely. Finally, the separated flap was inset immediately into the donor bed and sutured using 4-0 nonabsorbable silk. The random flap area was divided into the proximal (area I), intermediate (area II), and distal (area III) zones, each of the same size. On day 7, all mice were euthanized with an overdose of pentobarbital sodium. Six mice in each group were killed for Western blotting, and six rats in each group were killed for immunofluorescence, immunohistochemistry (IHC), and H&E staining. Six mice in each group were used for the general evaluation of survival, the tissue edema assessment, and laser Doppler blood flow imaging.

### Drug Administration

A 2% dimethyl sulfoxide (DMSO)–saline solution was used as the solvent for the intraperitoneal injections (Alawi et al., 2015; Gao et al., 2018; Hallajian et al., 2018). No obvious toxic effects were reported. Therefore, BA was dissolved in 2% DMSO in normal saline to a concentration of 50 mg/mL. Each mouse in the BA group received BA (20 mg/kg per day) by intraperitoneal injection for 7 days after the operation. The BA + 3MA group was treated with 3MA (15 mg/kg) 30 min before BA administration (dose) every time. Mice in the control group received an equal volume of DMSO–saline solution (vehicle control) for 7 days. All animals were euthanized by pentobarbital sodium (overdose), and histological samples were harvested.

### General Evaluation of Flap Survival

Macroscopic development and characteristics of texture, appearance, color, and hair condition of the flaps were observed for 7 days after the surgery. On postoperative days 3 and 7, photographs of the random flap were acquired to estimate flap viability. All photographs were measured using Image-Pro Plus imaging software (version 6.0; Media Cybernetics, Silver Spring, MD, USA) to calculate the surviving area, and the percentage of viable area was measured as follows: the extent of viable area × 100%/total area.

### Assessment of Tissue Edema

Tissue edema is an important factor involved in necrosis of ischemic flaps. Thus, the extent of edema is a crucial indicator of the tendency for necrosis. Tissue edema was reflected according to the water content of the flaps. On postoperative day 7, six flap tissue samples from each group were weighed, dehydrated in an autoclave at 50°C, and weighed until weight remained stable for at least 2 days. The percentage of water was measured as [(wet weight − dry weight)/wet weight] × 100%.

### Laser Doppler Blood Flow Imaging

The blood supply and vascular flow to the flaps were evaluated by laser Doppler blood flow (LDBF) measurements (Abraham et al., 2016). On postoperative day 7, a laser Doppler instrument (Moor Instruments, Axminster, UK) was used to scan the mice in a safe environment under anesthesia. The protocol for the LDBF evaluation was according to a previous study (Lin et al., 2018a). The entire dorsal skin site, including the flap area, was scanned by laser Doppler. Laser Doppler blood flow commonly provides deeper penetration, which enables reinforced visualization of small vessels under the tissue surface, and is perfect for an angiogenesis assessment. Perfusion units were seen as indices of blood flow. The blood flow of the random-pattern skin flaps was quantified by LDI Review software (version 6.1; Moor Instruments, Wilmington, DE, USA). We measured each mouse three times and calculated the mean value.

### Hematoxylin and Eosin Staining

On postoperative day 7, six samples (1 × 1 cm) of the central area from flap area II were acquired and sampled after sacrifice. The specimens were fixed in 4% paraformaldehyde for 24 h and embedded in paraffin wax for transverse sectioning. Sections (4-mm thickness) were cut with a microtome and mounted on poly l-lysine–coated slides for H&E staining. We counted the number of microvessels per unit area (/mm^2^) to assess microvascular density (MVD) under a light microscope (×200 magnification; Olympus Corp., Tokyo, Japan).

### Immunohistochemistry

Six sections of the central part of area II in each group were deparaffinized in xylene and then rehydrated in a graded ethanol series. After washing, 3% hydrogen peroxide solution was added to the sections to block endogenous peroxidase. Then, 10.2 mM sodium citrate buffer (pH 6.0) was used for antigen retrieval (20 min, 95°C). After blocking with 10% (w/v) bovine serum albumin phosphate-buffered saline for 10 min, the sections were incubated with primary antibodies: CD34 (1:100), VEGF (1:200), cadherin 5 (1:200), CASP3 (1:200), SOD1 (1:100), and CTSD (1:100) overnight at 4°C. The slides were further incubated with HRP-conjugated second antibody (1:1,000), stained with a DAB detection kit, and counterstained using hematoxylin. Slides were imaged at 200× magnification using the DP2-TWAN image-acquisition system (Olympus). Integral absorbance of VEGF-, cadherin 5-, CASP3-, SOD1- and CTSD-, and CD34-positive blood vessels was calculated using Image-Pro Plus software (Media Cybernetics). We counted six random fields in three random sections of each tissue sample.

### Western Blotting

After the mice had been euthanized, samples (0.5 × 0.5 cm) from the middle of area II flaps (n = 6) in each group were harvested and stored at −80°C for Western blotting analyses. After extracting the flap tissues with lysis buffer, the proteins were measured using the BCA assay. An equal amount of 60 µg protein was separated by 12% (w/v) gel electrophoresis and transferred to polyvinylidene difluoride membranes (Roche Applied Science, Indianapolis, IN, USA). After blocking with 5% (w/v) nonfat milk for 2 h at room temperature, the membranes were incubated with the subsequent primary antibodies at 4°C overnight: VEGF (1:1,000), MMP-9 (1:1,000), cadherin 5 (1:1,000), HO1 (1:1,000), eNOS (1:1,000), SOD1 (1:1,000), Bax (1:1000), CYC (1:1,000), caspase 3 (CAPS3) (1:1,000), Beclin 1 (1:1,000), p62 (1:1,000), LC3 (1:500), VPS34 (1:1,000), CTSD (1:1,000), and GAPDH (1:1,000). Then the membranes were incubated with HRP-conjugated IgG secondary antibody (1:5,000) for 2 h at room temperature. The bands on the membranes were visualized using the ECL Plus Reagent Kit. Band intensity was quantified using Image Lab 3.0 software (Bio-Rad, Hercules, CA, USA).

### Immunofluorescence Staining

Six sections of area II in each group were deparaffinized in xylene and rehydrated in a graded ethanol series. After washing, the sections were treated with 10.2 mM sodium citrate buffer for antigen retrieval (20 min, 95°C). After permeabilizing with 0.1% (v/v) PBS-Triton X-100 (10 min) and blocking with 10% (v/v) bovine serum albumin in PBS (1 h), the slides were incubated at 4°C overnight with anti-LC3II monoclonal antibody (1:200). Next, we washed the sections three times for 10 min each at room temperature and incubated the sections with FITC-conjugated secondary antibody for 1 h at room temperature. The slides were evaluated under a fluorescence microscope (Olympus). The percentage of LC3II-positive cells in the dermal layer was measured by counting six random fields on three random sections from each tissue sample.

### Statistical Analyses

Statistical analyses were implemented using SPSS version 19 software (SPSS Inc., Chicago, IL, USA). All data are presented as mean ± standard error. Comparisons of mean values between two groups were performed using the independent-sample *t* test. *P* < 0.05 was considered significant.

## Results

### BA Promotes Survival of Random-Pattern Skin Flaps and Reduces Tissue Edema

**Figure 1:**
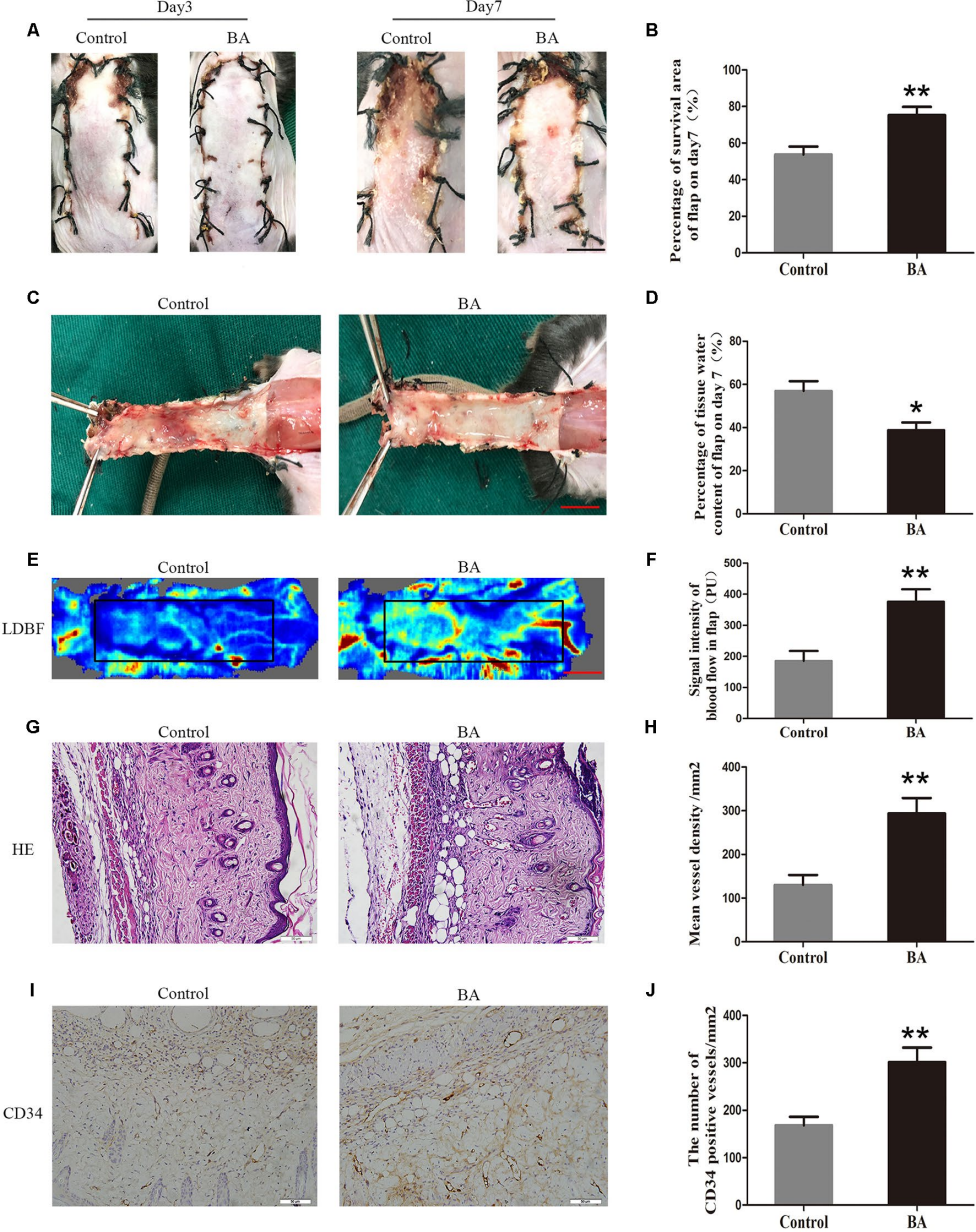
Betulinic acid (BA) promotes survival and reduces tissue edema in random-pattern skin flaps. **(A)** Digital photographs of the flaps in the control and BA groups were taken on postoperative days 3 and 7 (scale bar, 1.0 cm). **(B)** Histogram of the percentage of survival area on postoperative day 7. **(C)** Digital photographs of the tissue edema in the control and BA groups on postoperative day 7 (scale bar, 1.0 cm). **(D)** Histogram of the percentage of tissue water content. **(E)** Laser Doppler blood flow images of flaps on day 7 showing vascular flow and blood supply (scale bar, 1.0 cm). **(F)** Histogram of the blood flow signal intensity of the flaps. **(G)** Hematoxylin and eosin (H&E)–stained area II of the flaps in each group showing the vessels (original magnification ×200; scale bar, 50 µm). **(H)** Histogram of the percentage of MVD according to H&E staining in each group. **(I)** Immunohistochemistry for CD34 to mark vessels of area II of the skin flaps in the control and BA groups (original magnification ×200; scale bar, 50 µm). **(J)** Histogram of the percentage of CD34-positive vessel density. Data are mean ± standard error, n = 6 per group. *P < 0.05 and **P < 0.01, vs. control group.

On postoperative day 3, the appearance of the skin flaps in both groups was pale and swollen in area III without visible necrosis in area III. No significant differences in flap survival area were observed between the two groups (**Figure 1A**). On postoperative day 7, both groups exhibited survival in area I, whereas the necrosis in area III had become darker and spread to area II, with scabbing and hardening (**Figure 1A**). The difference in survival area between each group was more obvious than on day 3, as the BA group had a significantly larger mean survival area than the control group (75.32 ± 4.50% and 53.68 ± 4.41%, respectively; *P* = 0.006; **Figure 1B**). To observe the extent of edema, flap tissues from both groups were harvested to look at the inner side. As depicted in **Figure 1C**, the distal section of the flaps was edematous and bruised, with subcutaneous venous blood congestion in the control group. These signs were less visible in the BA group compared to the control group. Mean water content in the BA group flaps was much lower than that in the control group (38.78 ± 3.60% and 56.97 ± 4.53%, respectively; *P* = 0.010; **Figure 1D**), which implies that tissue edema was alleviated by the BA treatment. In addition, the BA group revealed more apparent blood flow signal intensity in random-pattern skin flaps according to the LDBF pictures than the control group (**Figure 1E**). After data processing, the blood flow signal intensity by LDBF imaging was enhanced in the BA group compared to the control group (376.02 ± 39.83 PU and 185.40 ± 31.84 PU, respectively; *P* = 0.004; **Figure 1F**). As shown in **Figure 1G**, the BA group generated more microvessels than the control group. We calculated mean MVD to reflect angiogenesis from the H&E staining observations. The MVD of area II in the BA group was significantly higher than that in the control group (293.75 ± 35.34/mm^2^ and 130.20 ± 22.76/mm^2^; *P* = 0.003; **Figures 1G, H**). For further quantification of MVD, we used CD34 IHC to mark the ECs in the vessels. Immunohistochemistry revealed that the number of CD34-positive vessels increased in the BA group compared to the control group (301.52 ± 30.61/mm^2^ and 168.30 ± 17.95/mm^2^, respectively; *P* ≤ 0.004; **Figures 1I, J**).

### BA Enhances Angiogenesis in the Ischemic Area of Flaps

**Figure 2:**
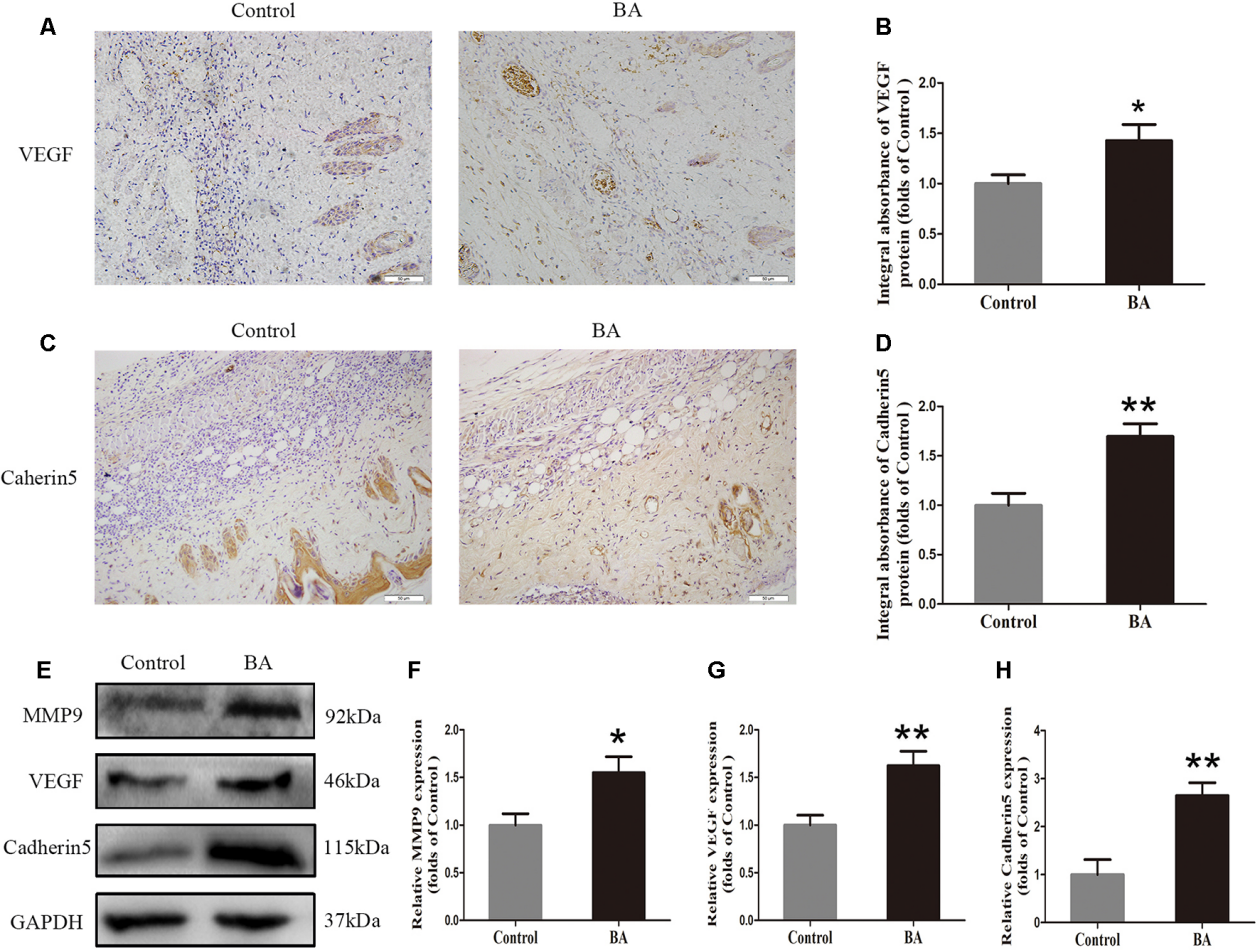
Betulinic acid (BA) enhances angiogenesis in the ischemic area of the flaps. **(A, C)** Immunohistochemistry (IHC) for vascular endothelial growth factor (VEGF) and cadherin 5 expression in the flaps of the control and BA groups (original magnification ×200; scale bar, 50 µm). **(B, D)** Histograms of optical density values for VEGF and cadherin 5 by IHC in each group. **(E)** Western blotting of the levels of VEGF, matrix metalloproteinase 9 (MMP9), and cadherin 5 in the control and BA groups. Gels were electrophoresed under the same experimental conditions, and cropped blots are shown here. **(F–H)** Histograms of optical density values for MMP9, VEGF, and cadherin 5 as calculated by Western blotting. Data are mean ± standard error, n = 6 per group. *P < 0.05 and **P < 0.01, vs. control group.

Vascular endothelial growth factor, MMP9, and cadherin 5 expression was assessed by IHC. As depicted in **Figure 2A**, VEGF expression in vessels and stromal cells was higher in the Sal B group than in the control group (*P* = 0.004; **Figure 2B**). Cadherin 5, a protein that is mainly detected in vessels and stromal cells (**Figure 2C**), was also significantly higher in the BA group than in the control group (*P* = 0.003; **Figure 2D**). The expression of angiogenesis-related proteins, such as MMP9, VEGF, and cadherin 5, in area II of the random-pattern skin flaps, was evaluated by Western blotting (**Figure 2E**). The optical density values of all of these proteins increased in the BA group compared to the control group (*P* = 0.023, *P* = 0.007, and *P* = 0.003, respectively; **Figures 2F–H**).

### BA Alleviates Apoptosis in the Ischemic Area of Flaps

**Figure 3:**
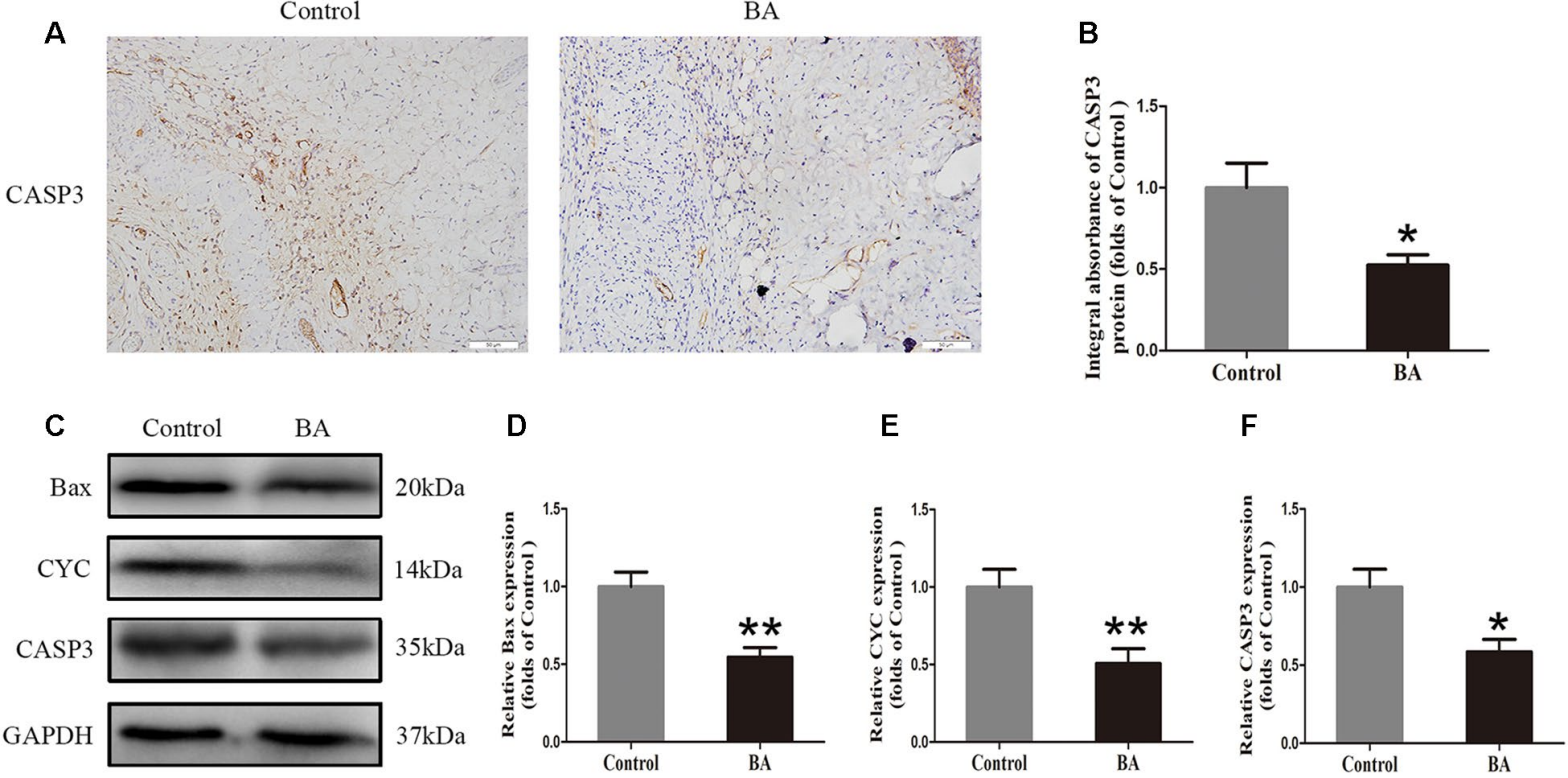
Betulinic acid (BA) alleviates apoptosis in the ischemic area of the flaps. **(A)** Caspase 3 (CASP3) levels of the flaps in the control and BA groups as assessed by immunohistochemistry (IHC) (original magnification ×200; scale bar, 50 µm). **(B)** Histograms of optical density values for CASP3 by IHC in each group. **(C)** Western blotting for Bax, CYC, and CASP3 expression in the skin flaps of the control and BA groups. Gels were electrophoresed under the same experimental conditions, and cropped blots are shown here. **(D–F)** Histograms of the optical density values of matrix metalloproteinase 9 (MMP9), vascular endothelial growth factor (VEGF), and cadherin 5 in the two groups as measured using Western blotting. Data are mean ± standard error, n = 6 per group. *P < 0.05 and **P < 0.01, vs. control group.

Immunohistochemistry was performed to measure the CASP3 level in the dermis layer of area II in the control and BA groups. The CASP3 level in vessels and stromal cells of the BA group was lower than that in the control group (**Figure 3A**), and so is the integral absorbance of CAPS3 in the BA group (*P* = 0.016; **Figure 3B**). Western blotting was implemented to determine the expression of Bax, CYC, and CASP3 in the skin flaps (**Figure 3C**). The BA group expressed less Bax, CYC, and CASP3 than the control group (*P* = 0.002, *P* = 0.008, and *P* = 0.014, respectively; **Figures 3D–F**).

### BA Attenuates Oxidative Stress in the Ischemic Area of Flaps

**Figure 4:**
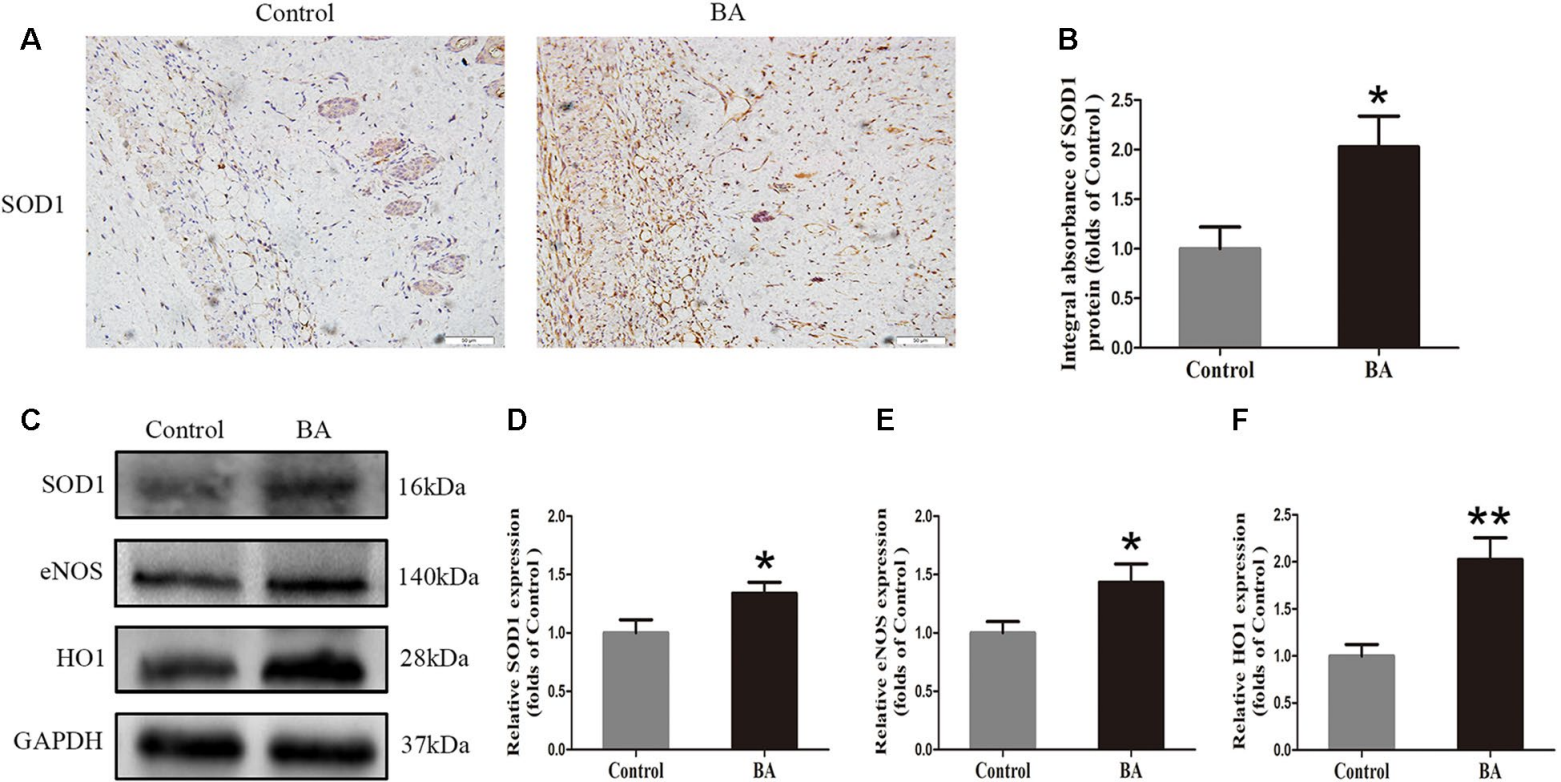
Betulinic acid (BA) attenuates oxidative stress in the ischemic area of the flaps. **(A)** Immunohistochemistry (IHC) for superoxide dismutase 1 (SOD1) expression in the flaps of each group (original magnification ×200; scale bar, 50 µm). **(B)** Histograms of optical density values of SOD1 by IHC in each group. **(C)** Western blotting for SOD1, endothelial nitric oxide synthase (eNOS), and heme oxidase 1 (HO1) protein expression in the skin flaps. Gels were electrophoresed under the same experimental conditions, and cropped blots are shown here. **(D–F) **Histograms of the optical density values of matrix metalloproteinase 9 (MMP9), vascular endothelial growth factor (VEGF), and cadherin 5 in the two groups as evaluated by Western blotting. Data are means ± standard error, n = 6 per group. *P < 0.05 and **P < 0.01, vs. control group.

To verify the effects of BA on oxidative stress in skin flaps, we implemented IHC to determine SOD1 status, which reflected the magnitude of the oxidative stress in area II of the flaps. As shown in **Figure 4A**, a higher SOD1 level and integral absorbance in the dermis were detected in the BA group than in the control group (*P* = 0.022; **Figure 4B**). Western blotting was performed to measure the status of SOD1, eNOS, and HO1 in the skin flaps (**Figure 4C**). The optical density values of these proteins were significantly upregulated in the BA group compared to the control group (*P* = 0.042, *P* = 0.041, and *P* = 0.003, respectively; **Figures 4D–F**).

### BA Upregulates Autophagy in the Ischemic Area of Flaps

**Figure 5:**
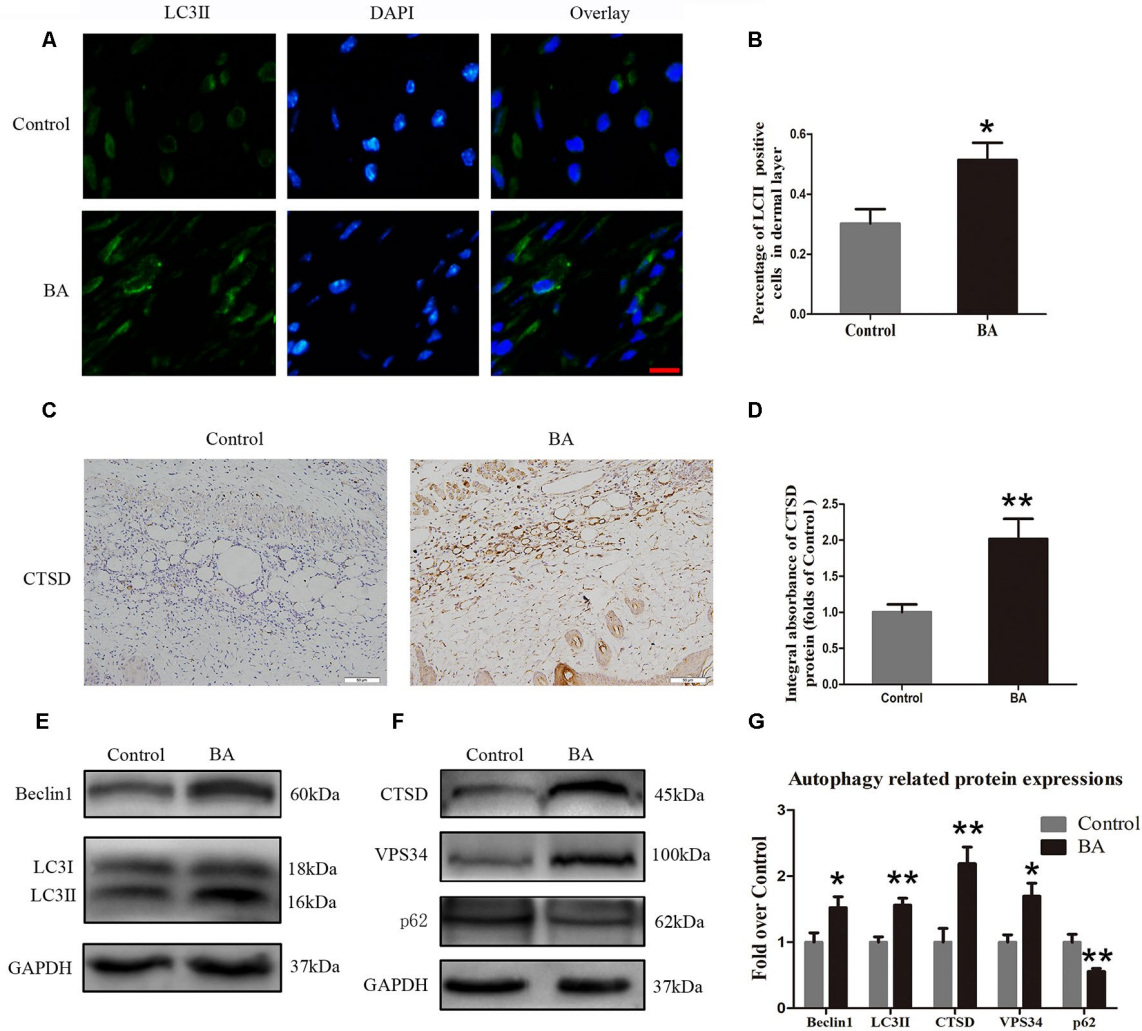
Betulinic acid (BA) upregulates autophagy in the ischemic area of the flaps. **(A) **Immunofluorescence to determine the LC3II expression level was performed to show the autophagosomes in the cells of the skin flaps: autophagosomes (green) in the dermis in area II; nuclei counterstained with DAPI (blue) (scale bar: 50 mm). **(B)** Histogram of the percentage of LC3II-positive cells. **(C)** CTSD level in the flaps from each group by immunohistochemistry (IHC) (original magnification ×200; scale bar, 50 µm). **(D)** Histograms of optical density values for CTSD by IHC in each group. **(E, F)** Western blotting of the Beclin 1, LC3II, CTSD, VPS34, and p62 proteins in the skin flaps of the control and BA groups. Gels were electrophoresed under the same experimental conditions, and cropped blots are shown here. **(G)** Histograms of the optical density values of Beclin 1, LC3II, CTSD, VPS34, and p62 in the two groups as determined by Western blotting. Data are mean ± standard error, n = 6 per group. *P < 0.05 and **P < 0.01, vs. control group.

Beclin 1 and VPS34 are localized to the preautophagosomal structure, LC3II is localized in the autophagosome membrane, CTSD is a marker of autolysosomes, and p62 can be used to monitor autophagic degradation. Hence, we assessed the levels of Beclin 1, VPS34, LC3II, CTSD, and p62. We carried out immunofluorescence to determine LC3II expression, which indicates autophagosomes in the cells of flap area II. As depicted in **Figure 5A**, we marked autophagosomes with LC3II punctate dots (green) and labeled the nuclei with DAPI (blue). A high percentage of LC3II-positive cells was detected in the dermis of the BA group compared to the control group (**Figure 5B**). Cathepsin D status in the dermis of the BA group was higher than that in the control group (**Figure 5C**) and the CTSD integral absorbance of the BA group (*P* = 0.007; **Figure 5D**). We also implemented Western blotting to determine Beclin 1, VPS34, LC3II, CTSD, and p62 levels in the skin flaps (**Figures 5E, F**). The expression of Beclin 1, LC3II, VPS34, and CTSD was significantly higher in the BA group than in the control group (*P* = 0.036, *P* = 0.02, *P* = 0.012, and *P* = 0.005, respectively; **Figure 5G**), whereas p62 expression was lower in the BA group than that in the control group (*P* = 0.006; **Figure 5G**).

### Inhibiting Autophagy Reverses the Effects of BA on Angiogenesis, Apoptosis, and Oxidative Stress

**Figure 6:**
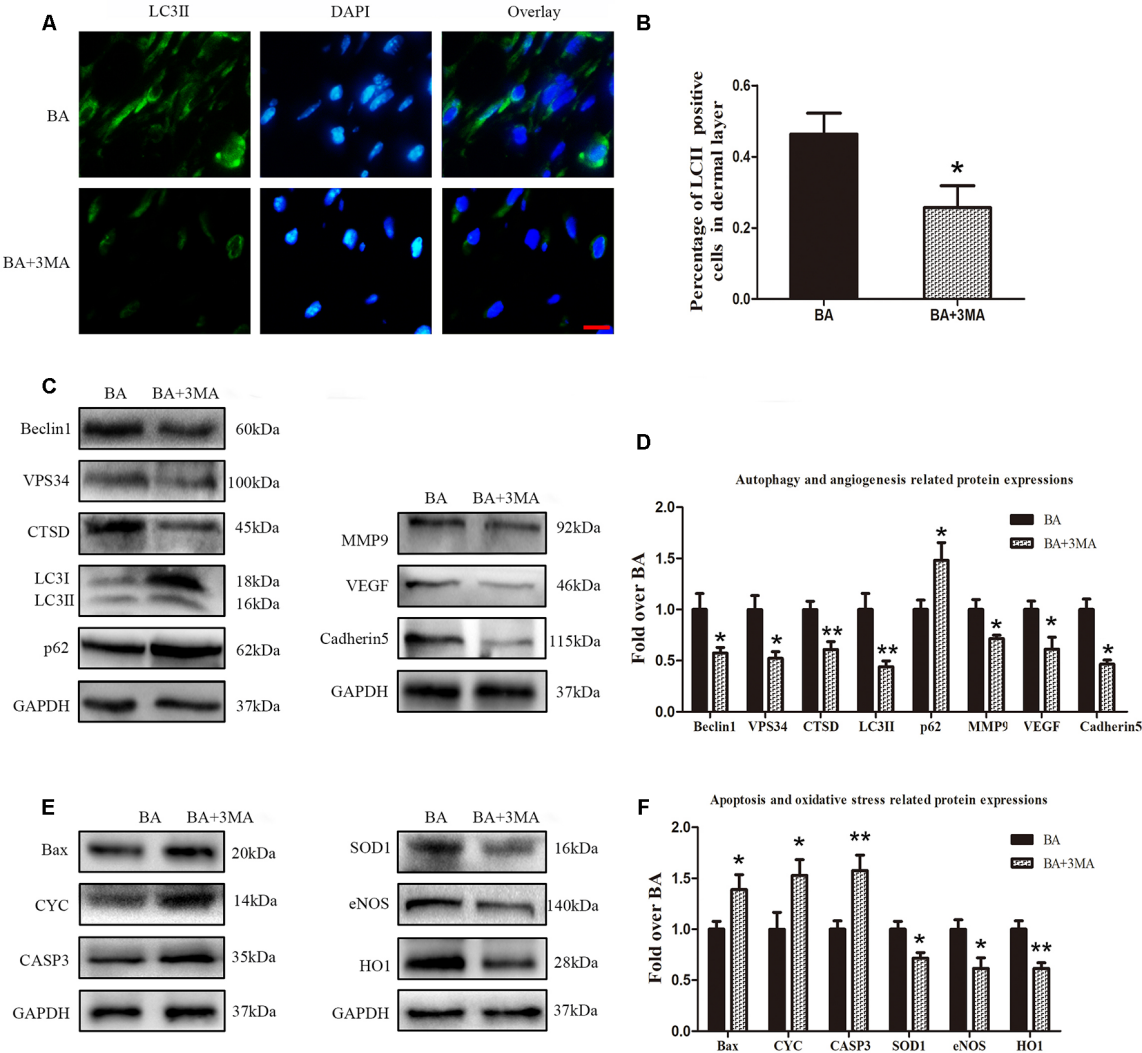
Suppression of autophagy reverses the effects of betulinic acid (BA) on angiogenesis, apoptosis, and oxidative stress. **(A)** Immunofluorescence was performed to determine the LC3II expression level to show the autophagosomes in the cells of the skin flaps: autophagosomes (green) in the dermis in area II; nuclei counterstained with DAPI (blue) (scale bar: 50 mm). **(B)** Histogram of the frequency of LC3II-positive cells. **(C**, **E)** Western blotting for the expression of the autophagy-related proteins VPS34, p62, LC3II, Beclin 1, and CTSD; angiogenesis-related protein vascular endothelial growth factor (VEGF), cadherin 5, and matrix metalloproteinase 9 (MMP9); the oxidative stress–related proteins superoxide dismutase 1 (SOD1), heme oxygenase 1 (HO1), and endothelial nitric oxide synthase (eNOS); and the apoptosis-related proteins Bax, CYC, and CASP3. Gels were electrophoresed under the same experimental conditions, and cropped blots are shown here. **(D**, **F)** Histograms of optical density values of VPS34, p62, LC3II, Beclin 1, CTSD, VEGF, cadherin 5, MMP9, SOD1, HO1, eNOS, Bax, CYC, and CASP3 in the two groups as measured using Western blotting. Data are mean ± standard error, n = 6 per group. *P < 0.05 and **P < 0.01, vs. control group.

To demonstrate that autophagy is the major mechanism underlying the effects of BA, we further inspected BA activity in random-pattern skin flaps after treatment with the autophagy inhibitor 3MA. Immunofluorescence was performed to determine LC3II expression and indicate the autophagosomes in area II of the flaps (**Figure 6A**). A higher frequency of LC3II-positive cells was detected in the dermis of the BA group than the BA + 3MA group (**Figure 6B**). Western blotting showed that the status of Beclin 1, VPS34, CTSD, and LC3II was significantly higher in the BA group than that in the BA + 3MA group (*P* = 0.028, *P* = 0.01, *P* = 0.006, and *P* = 0.007, respectively; **Figures 6C, D**), as were those of MMP9, VEGF, cadherin 5 (*P* = 0.021, *P* = 0.022, and *P* = 0.001, respectively; **Figures 6C, D**) and SOD1, eNOS, and HO1 (*P* = 0.013, 0.02, and 0.003, respectively; **Figures 6E, F**), whereas lower expression of p62, CYC, Bax, and CAPS3 was observed in the BA group (*p* = 0.035, *P* = 0.042, *P* = 0.042, and *P* = 0.008, respectively; **Figures 6C–F**).

### Suppressing Autophagy Reverses the Effects of BA on Flap Viability

**Figure 7:**
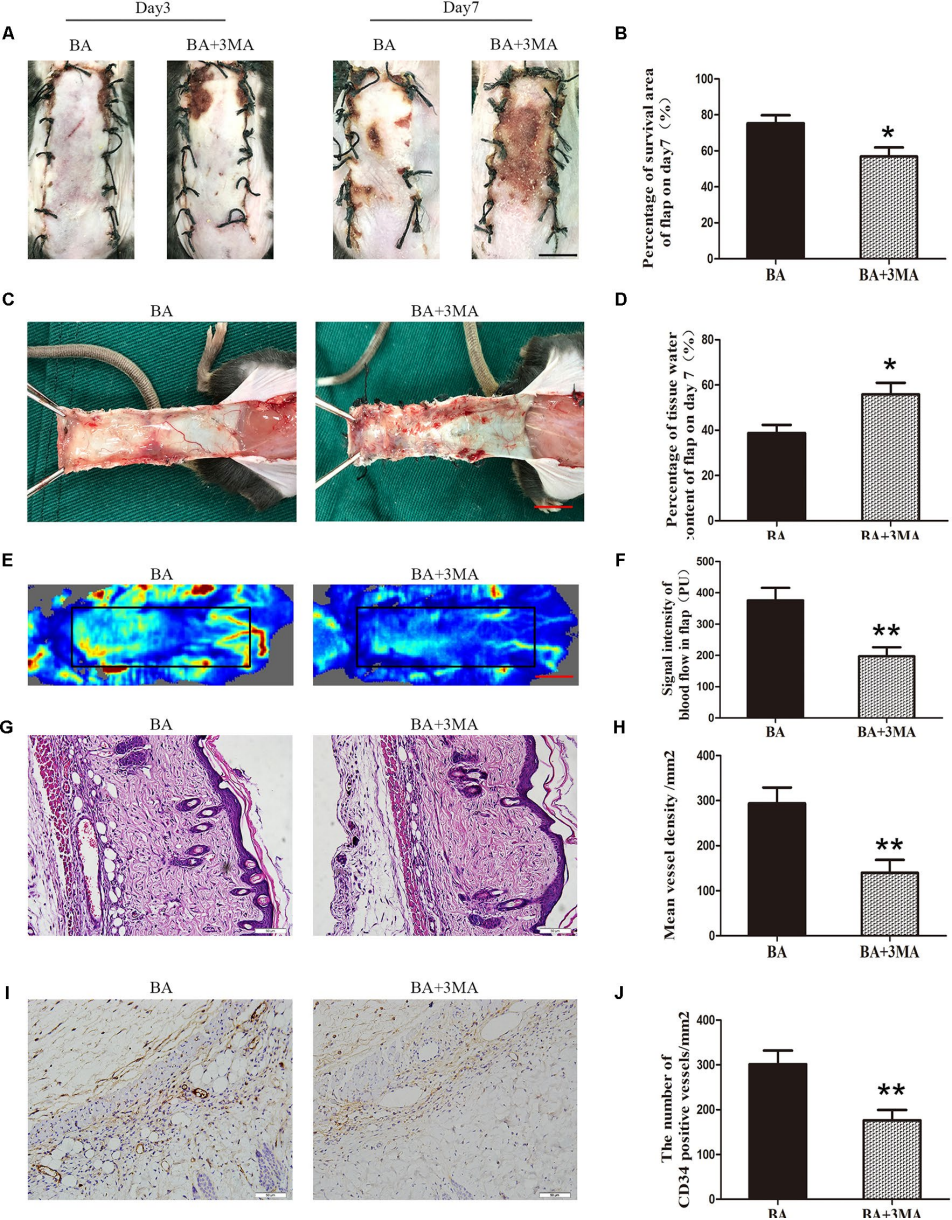
Inhibition of autophagy reverses the effects of betulinic acid (BA) on flap vitality. **(A)** Digital photographs of flaps from the BA and BA + 3-methyladenine (3MA) groups were taken on postoperative days 3 and 7 (scale bar, 1.0 cm). **(B)** Histogram of the percentage survival area on postoperative day 7. **(C)** Digital photographs of the tissue edema in the BA and BA + 3MA groups on postoperative day 7 (scale bar, 1.0 cm). **(D)** Histogram of the percentage of water content in the flap tissues. **(E)** Laser Doppler blood flow images of flaps on day 7 showing vascular flow and blood supply (scale bar, 1.0 cm). **(F)** Histogram of the blood flow signal intensity in the flaps. **(G)** Hematoxylin and eosin (H&E)–stained area II of the flaps in the each group to show vessels (original magnification ×200; scale bar, 50 µm). **(H)** Histogram of the percentage of MVD according to H&E staining in the BA and BA + 3MA groups. **(I)** Immunohistochemistry for CD34 to mark vessels of area III on the skin flaps from the BA group and the BA + 3MA group (original magnification ×200; scale bar, 50 µm). **(J)** Histogram of the percentage of CD34-positive vessel density. Data are mean ± standard error, n = 6 per group. *P < 0.05 and **P < 0.01, vs. control group.

On postoperative day 3, no significant differences were seen in flap survival area between the BA and BA + 3MA groups (**Figure 7A**). On day 7 after the operation, necrosis in area III became darker and spread to area II (**Figure 7A**). The difference in the survival area between the groups was more apparent than before, with the BA group revealing significantly higher mean survival area than the BA + 3MA group (75.32 ± 4.50% and 56.85 ± 5.02%, respectively; *P* = 0.021; **Figure 7B**). The distal part on the inner side of the flap was swollen and bruised, with subcutaneous venous blood stasis in the BA + 3MA group (**Figure 7C**). Mean tissue water content in the BA group was significantly lower than that in the BA + 3MA group (38.78 ± 3.60% and 55.82 ± 5.16%, respectively; *P* = 0.022; **Figure 7D**). Moreover, as shown in **Figure 7E**, the BA group had stronger blood flow signal intensity in the skin flaps than that in the BA + 3MA group. After data quantification, the BA group revealed larger blood flow signal intensity than that in the BA + 3MA group (376.02 ± 39.83 PU and 197.43 ± 28.66 PU, respectively; *P* = 0.005; **Figure 7F**). Hematoxylin and eosin staining was performed to determine the number of microvessels. The BA group (293.75 ± 35.34/mm^2^) had a greater number of microvessels than the BA + 3MA group (139.82 ± 28.68/mm^2^; *P* = 0.007; **Figures 7G, H**). Finally, as shown in **Figure 7I**, the number of CD34-positive vessels (176.30 ± 23.10/mm^2^) decreased in the BA + 3MA group compared to the BA group (301.52 ± 30.61/mm^2^; *P* = 0.009; **Figure 7J**).

## Discussion

Betulinic acid is a natural plant triterpenoid, which is distributed worldwide (Xie et al., 2017; Sarkaki et al., 2018). This agent has attracted attention because recent studies have demonstrated its benefit in the treatment of various diseases, involving myocardial IRI, liver fibrosis, Alzheimer disease, and diabetes and diabetic complications by protecting the microcirculation, promoting autophagy, and inhibiting oxidative stress and cell apoptosis (Genet et al., 2010; Xie et al., 2017; Liu et al., 2018; Sarkaki et al., 2018; Wang et al., 2018). Necrosis of the skin flap is a common postoperative complication, due to inadequate blood supply and IRI. Whether BA protects the flap from ischemia remains unknown. Our study revealed that BA increased survival of random-pattern skin flaps, and the underlying mechanism involved increasing neovascularization and autophagy, as well as dampening oxidative stress and apoptosis.

Betulinic acid protects microcirculation in Alzheimer disease (Sarkaki et al., 2018) and improves cerebral blood flow in rats with vascular dementia (Kaundal et al., 2018). In our study, the number of microvessels, reflected by H&E and IHC staining for CD34, was apparently enhanced in the BA group. Furthermore, blood flow as observed by LDBF was higher in the BA group than in the control group. Our findings reveal that treatment with BA enhanced the survival of skin flaps by promoting the blood supply and angiogenesis, which was mainly stimulated by hypoxia in ischemic tissue. New blood vessels formed from preexisting cell connections through mitosis, sprouting, proliferation, and migration of ECs and growth of new capillaries (Longchamp et al., 2018; Nowak-Sliwinska et al., 2018; Zhang et al., 2018). Matrix metallopeptidase 9 enhances tissue remodeling by degrading the extracellular matrix and plays an important role in angiogenesis (Mărginean et al., 2019). Vascular endothelial growth factor promotes EC migration, proliferation, and vessel formation by acting on ECs and combining with the cell-surface tyrosine kinase receptor VEGFR2, initiating an orchestrated cascade of signal transduction *via* the PI3K and MAPK pathways (Longchamp et al., 2018). Vascular endothelial cadherin (VE-cadherin/cadherin 5) is specifically expressed in adherens junctions of ECs where it plays a significant role in cell–cell adhesion and signal transduction (Ahrens et al., 2003). Our IHC and Western blotting results revealed that VEGF and cadherin 5 expression in vessels and stromal cells in the dermis was upregulated after the BA treatment. The results also showed that BA strongly induced increases in the cadherin 5 protein. Taken together, we hypothesized that BA facilitates neovascularization in the dermis of skin flaps by upregulating VEGF, MMP9, and cadherin 5.

Partial or total necrosis of the flap is caused by IRI (Gurlek et al., 2006). During reperfusion, the accumulation of ROS destroys the structure of cell membranes, nucleic acids, and chromosomes, triggering oxidative stress and apoptosis (Yao et al., 2018). Reactive oxygen species destroy cell membranes through lipid peroxidation, followed by destruction of the cell and accelerated cell death. Antioxidant enzymes, such as SOD, are the first-line cellular defense against oxidative injury (Kumar et al., 2016). Heme oxygenase 1 and eNOS also have antioxidant activity (Sun et al., 2018). Pretreatment with BA decreases oxidative stress, effectively alleviating remifentanil-induced hyperalgesia (Lv et al., 2018). The IHC and Western blotting results demonstrated that SOD1, eNOS, and HO1 expression in the dermis of skin flaps was enhanced in the BA group. Taken together, BA protected against IRI and oxidative stress.

Betulinic acid reportedly protects mice from cadmium chloride–induced toxicity by attenuating apoptosis in the kidneys and liver (Fan et al., 2018). Apoptosis is considered an important factor in IRI of skin flaps (Burns et al., 1998). Consequently, we hypothesized that BA downregulates apoptosis in the dermis of the random-pattern skin flaps, promoting survival of the flaps. We examined the level of apoptosis in the ischemic area of the flaps. Apoptosis is a highly conserved programmed cell death process that can be triggered by intrinsic (ROS or mitochondrial damage) or extrinsic (by growth factor withdrawal) factors (Fuchs and Steller, 2015). Reactive oxygen species quickly raise the permeability of the inner mitochondrial membrane and break the balance of Bcl-2–like antiapoptotic factors and Bax-like proapoptotic factors, leading to mitochondrial outer membrane permeabilization and the release of CYC, which initiates apoptosis (Fuchs and Steller, 2015; Chen et al., 2017). Once CYC is translocated to the cytoplasm, a high-molecular-weight compound is produced, which directly activates CAPS3, an executioner that triggers the cellular processes leading to apoptosis (Jiang and Wang, 2000; Li et al., 2015a; Rahman et al., 2015). Therefore, we examined Bax, CYC, and CASP3 expression levels to determine the extent of apoptosis. Immunohistochemistry and Western blotting revealed that BA apparently inhibited the level of Bax, CYC, and CASP3. In conclusion, BA attenuated apoptosis in the dermis of the skin flaps.

Autophagy is a highly conserved self-degradative process in which cytoplasmic materials, including organelles, are wrapped up by a double-membrane vesicle, called an autophagosome, for degradation (Wang et al., 2014b). Autophagy is an adaptation to starvation that is antiaging and antitumorigenic and prevents neurodegeneration, degradation of invading microorganisms, and presentation of intracellular antigens; therefore, autophagy plays an important role in the survival of mammalian cells (Itakura and Mizushima, 2010). Increasing evidence demonstrates that autophagy is beneficial in cardiovascular disease (Xie et al., 2011). For example, VEGF-A may induce angiogenesis after acute myocardial infarction *via* endoplasmic reticulum stress–mediated autophagy (Zou et al., 2019). Previous studies have reported that BA enhances the level of autophagy *via* the MAPK signaling pathway (Liu et al., 2018). This is the first study on BA-mediated induction of autophagy in random-pattern skin flaps. Autophagy involves formation of the autophagosome, fusion of the autophagosome with the lysosome, and digestion of the substrate. Consequently, we detected Beclin 1, LC3II, and VPS34 as indicators of autophagosomes (Glick et al., 2010): CTSD, a marker of autolysosomes (Kanamori et al., 2011), and p62, a marker of autophagic degradation (Zaffagnini et al., 2018). A high percentage of LC3II-positive cells in the dermis was discovered after the BA treatment. Cathepsin D status in the dermis of the BA group was higher than that in the control group, and so is the integral absorbance of CTSD. Western blotting showed that the expression of Beclin 1, LC3II, and VPS34 was significantly higher in the BA group, suggesting the formation of additional autophagosomes in the random-pattern skin flaps. Furthermore, a higher expression level of CTSD was detected in the flaps of the BA group, with lower levels of p62, indicating increased autophagic flux in the BA group. These results suggest that BA facilitates autophagy in random-pattern skin flaps.

The role of autophagy is a “double-edged sword” in cell survival. Autophagy is activated for cell survival in many diseases, such as acute myocardial infarction, but excessive activation of autophagy may trigger cell death (Zou et al., 2019). Therefore, we assessed the effects of BA-mediated autophagy in random-pattern skin flaps. 3-Methyladenine is a widely used inhibitor of autophagy because of its inhibitory effect on a PI3K, which is present in all eukaryotic cells and has essential roles in the formation of autophagosomes (Miller et al., 2010; Wang et al., 2016). The present study demonstrated that suppressing autophagy with 3MA reverses BA-mediated enhancement of flap vitality, a decrease in tissue edema, and increased MVD. Activating autophagy promotes angiogenesis after acute myocardial infarction in vascular ECs (Zou et al., 2019). We discovered that treatment with 3MA apparently decreased MMP9, VEGF, and cadherin 5 expression. Hence, we suspect that BA facilitates angiogenesis in skin flaps by inducing autophagy. Induction of autophagy protects against nutrient deprivation–induced mitochondrial apoptosis (Miyazaki et al., 2015). Our data demonstrate that Bax, CYC, and CASP3 expression decreased after the 3MA treatment, indicating that the antiapoptosis function of BA is mediated through the induction of autophagy. Upregulating autophagy offers cytoprotection against oxidative stress by degrading dysfunctional and damaged mitochondria and ubiquitinated proteins (Dutta et al., 2013). 3-Methyladenine clearly dampened the levels of SOD1, eNOS, and HO1. Consequently, BA alleviated oxidative stress in random-pattern skin flaps by activating autophagy.

Taken together, our findings show that BA enhances the survival of random-pattern skin flaps by promoting angiogenesis, dampening apoptosis, and reducing oxidative stress, which are all mediated by autophagy.

## Data Availability

The raw data supporting the conclusions of this manuscript will be made available by the authors, without undue reservation, to any qualified researcher.

## Ethics Statement

The animal study was reviewed and approved by Animal Research Committee of Wenzhou Medical University (wydw2017–0022).

## Author Contributions

JL and GB wrote the manuscript text. JL, EA, JD, SL, SS and ZS prepared figures and collected samples. ZJ, CZ and ZL analyzed data, WG and KZ designed the experiment. EA, HX, WG and KZ revised the manuscript. All authors reviewed and approved the final manuscript.

## Funding

This work was supported by grants from Natural Science Foundation of China (no. 81601705 to KZ, no. 81873942 to WG, no. 81801930 to JD, no. 81572227 and no. 81873992 to HX); Zhejiang Provincial Medicine and Health Technology Project (no. 2017KY472 to KZ); and Wenzhou Science and Technology Bureau Foundation (no. 2016Y0350 to JD).

## Conflict of Interest Statement

The authors declare that the research was conducted in the absence of any commercial or financial relationships that could be construed as a potential conflict of interest.

## Abbreviations

LDBF, laser Doppler blood flow; BA, betulinic acid; cadherin 5, vascular endothelial cadherin; CAPS3, caspase 3; CTSD, cathepsin D; CYC, cytochrome *c*; DAPI, 4′,6-diamidino-2-phenylindole; eNOS, endothelial nitric oxide synthase; FITC, fluorescein isothiocyanate; GAPDH, glyceraldehyde 3-phosphate dehydrogenase; H&E, hematoxylin and eosin; HO1, heme oxygenase 1; HRP, horseradish peroxidase; IHC, immunohistochemistry; LC3B, autophagy marker light chain 3B; 3MA, 3-methyladenine; MMP9, matrix metallopeptidase 9; MVD, microvascular density; IRI, ischemia–reperfusion injury; SOD, superoxide dismutase; VEGF, vascular endothelial growth factor; VPS34, phosphoinositide-3-kinase.
